# Outcomes, Healthcare Resource Utilization, and Costs of Overall, Community-Acquired, and Hospital-Acquired Acute Kidney Injury in COVID-19 Patients

**DOI:** 10.36469/001c.57651

**Published:** 2023-02-23

**Authors:** Jay L. Koyner, Rachel H. Mackey, Ning A. Rosenthal, Leslie A. Carabuena, J. Patrick Kampf, Paul McPherson, Toni Rodriguez, Aarti Sanghani, Julien Textoris

**Affiliations:** 1 Section of Nephrology University of Chicago, Chicago, Illinois; 2 Premier, Inc., PINC AI Applied Sciences, Charlotte, North Carolina; 3 Department of Epidemiology University of Pittsburgh School of Public Health, Pittsburgh, Pennsylvania; 4 Astute Medical Inc. (a bioMerieux company), San Diego, California; 5 Global Medical Affairs bioMerieux, Inc., Durham, North Carolina; 6 bioMerieux, Inc., Global Medical Affairs, Durham, North Carolina; 7 bioMerieux, SA, Global Medical Affairs, Lyons, France; 8 Service d´Anesthésie et de Réanimation, Lyons, France

**Keywords:** COVID-19, acute kidney injury (AKI), hospital-acquired AKI, community-acquired AKI, healthcare resource utilization, costs, clinical outcomes

## Abstract

**Background:** In hospitalized patients with COVID-19, acute kidney injury (AKI) is associated with higher mortality, but data are lacking on healthcare resource utilization (HRU) and costs related to AKI, community-acquired AKI (CA-AKI), and hospital-acquired AKI (HA-AKI). **Objectives:** To quantify the burden of AKI, CA-AKI, and HA-AKI among inpatients with COVID-19. **Methods:** This retrospective cohort study included inpatients with COVID-19 discharged from US hospitals in the Premier PINC AI™ Healthcare Database April 1–October 31, 2020, categorized as AKI, CA-AKI, HA-AKI, or no AKI by ICD-10-CM diagnosis codes. Outcomes were assessed during index (initial) hospitalization and 30 days postdischarge. **Results:** Among 208 583 COVID-19 inpatients, 30%, 25%, and 5% had AKI, CA-AKI, and HA-AKI, of whom 10%, 7%, and 23% received dialysis, respectively. Excess mortality, HRU, and costs were greater for HA-AKI than CA-AKI. In adjusted models, for patients with AKI vs no AKI and HA-AKI vs CA-AKI, odds ratios (ORs) (95% CI) were 3.70 (3.61-3.79) and 4.11 (3.92-4.31) for intensive care unit use and 3.52 (3.41-3.63) and 2.64 (2.52-2.78) for in-hospital mortality; mean length of stay (LOS) differences and LOS ratios (95% CI) were 1.8 days and 1.24 (1.23-1.25) and 5.1 days and 1.57 (1.54-1.59); and mean cost differences and cost ratios were 7163and1.35(1.34−1.36)and19 127 and 1.78 (1.75-1.81) (all *P* < .001). During the 30 days postdischarge, readmission LOS was ≥6% longer for AKI vs no AKI and HA-AKI vs CA-AKI; outpatient costs were ≥41% higher for HA-AKI vs CA-AKI or no AKI. Only 30-day new dialysis (among patients without index hospitalization dialysis) had similar odds for HA-AKI vs CA-AKI (2.37-2.8 times higher for AKI, HA-AKI, or CA-AKI vs no AKI). **Discussion:** Among inpatients with COVID-19, HA-AKI had higher excess mortality, HRU, and costs than CA-AKI. Other studies suggest that interventions to prevent HA-AKI could decrease excess morbidity, HRU, and costs among inpatients with COVID-19. **Conclusions:** In adjusted models among COVID-19 inpatients, AKI, especially HA-AKI, was associated with significantly higher mortality, HRU, and costs during index admission, and higher dialysis and longer readmission LOS during the 30 days postdischarge. These findings support implementation of interventions to prevent HA-AKI in COVID-19 patients.

## BACKGROUND

Acute kidney injury (AKI) is an abrupt decrease in kidney function, defined and staged for severity based on decreased urine output and increases in serum creatinine relative to reference (baseline) creatinine levels for each patient.^1^ Prior to the COVID-19 pandemic, AKI occurred in 5% to 18% of adult hospitalized patients^2,3^ and 20% to 75% of those admitted to intensive care units (ICUs).^4–7^ Hospitalized patients with AKI have worse clinical outcomes,^3,5,7,8^ higher healthcare resource utilization (HRU),^3,9^ and approximately twofold higher costs than patients without AKI.^3,10^

AKI is now known to be a common complication of COVID-19, but it is unclear whether COVID-19 affects the kidneys directly, or indirectly, by causing more severe illness, similar to other severe infections.^11^ AKI may be more frequent and more severe (ie, more stage 3 AKI) in hospitalized patients with than without COVID-19, and is associated with 7 to 15 times higher mortality risk vs COVID-19 patients without AKI.^12–15^ Furthermore, in patients without COVID-19, AKI that first occurs in the hospital (hospital-acquired AKI [HA-AKI]) is associated with higher mortality,^16^ longer hospital length of stay (LOS), and higher costs^17^ than community-acquired AKI (CA-AKI), which occurs prior to hospital admission. However, in the few small studies among COVID-19 patients, results are mixed regarding whether mortality is increased for patients with HA-AKI vs CA-AKI.^18–22^ Finally, to date, no studies have evaluated costs of AKI, CA-AKI, or HA-AKI in patients with COVID-19.

Quantifying the excess burden of AKI, especially HA-AKI, could provide motivation for targeted interventions to prevent and manage AKI in COVID-19 inpatients.^23^ Several studies have evaluated the implementation of the Kidney Disease: Improving Global Outcomes (KDIGO) “bundle” of guideline-recommended renal-protective measures to treat and prevent AKI, including optimization of volume status and hemodynamics, functional hemodynamic monitoring, avoidance of nephrotoxic drugs, and prevention of hyperglycemia.^1^ In non-COVID-19 patients, early identification and use of the KDIGO bundle can reduce the incidence,^24–26^ severity,^27^ duration,^28^ and progression of AKI^29^ and hospital LOS^27,28^ and costs.^30,31^ For example, the Acute Kidney Outreach to Reduce Deterioration and Death (AKORDD) study found that the cost of an AKI alert and outreach intervention was lower than usual care.^31^ Also, the UK-based Tackling AKI study demonstrated that implementation of a multifaceted AKI intervention (e-alerts, care bundle, and an education program) resulted in shorter hospital LOS^28^ and lower costs for AKI patients.^30^ However, adherence to best practices (the KDIGO bundle) remains low among patients at high risk of AKI.^26,32,33^ Therefore, the objective of the current study was to evaluate the prevalence and incidence of AKI, CA-AKI, and HA-AKI and quantify associated clinical outcomes, healthcare resource utilization (HRU), and costs among adults hospitalized with COVID-19, to provide data to motivate improved prevention and management of AKI in COVID-19 patients.

## METHODS

### Data Source, Study Timeline, Cohort, and Exposure

We included patients with a COVID-19 discharge diagnosis from April 1, 2020, to October 31, 2020, from a US hospital in the Premier PINC AI™ Healthcare Database (PHD), formerly known as the Premier Healthcare Database, which has been widely used for published studies by the US Centers for Disease Control and Prevention and others.^34–37^ The PHD contains discharge information (eg, demographics, diagnosis and procedure codes, discharge status, and costs) from inpatient and hospital-based outpatient visits from more than 1000 geographically diverse hospitals and health systems. Masked identifiers track patients across inpatient and hospital-based outpatient settings within the same hospital/system. The PHD data are statistically de-identified and compliant with HIPAA (Health Insurance Portability and Accountability Act). Based on 45 CFR §46, the study was exempt from institutional review board approval, as in prior PHD studies. This study followed RECORD (REporting of studies Conducted using Observational Routinely collected health Data) guidelines, specifically for studies using administrative health data.

The first hospitalization with discharge from April 1, 2020, through October 31, 2020, that met inclusion and exclusion selection criteria (**Supplemental Figure S1**) was the index hospitalization. Inclusions were age 18 years and older and COVID-19 *International Classification of Diseases, Tenth Revision, Clinical Modification* (ICD-10-CM) diagnosis code U07.1. Exclusions, defined using ICD-10 diagnosis or procedure or current procedural terminology (CPT) codes (**Supplemental Table S1**), were renal transplantation or end-stage renal disease during index hospitalization or 365-day look-back period; more than 1 hospital inpatient or outpatient visits with dialysis-related ICD-10 diagnosis or procedure codes or CPT codes (**Supplemental Table S1**) during 365-day look-back period; or ICD-10 diagnosis code for stage 5 chronic kidney disease (CKD) (N18.5) with present on admission (POA) indicator = Yes.

The exposure, AKI, was defined by presence of ICD-10-CM diagnosis code N17.% during index hospitalization. AKI was subcategorized as CA-AKI, defined by POA = Yes, and HA-AKI, defined by POA = No (**Supplemental Methods**).

### Patient, Visit, Clinical, and Hospital Characteristics

Patient, visit, and hospital characteristics were evaluated during the index hospitalization. Patient demographics included age, sex, race/ethnicity, and primary payer. Hospital characteristics included size (number of beds), geographic region, urban vs rural, and teaching status. The ICD-10-CM diagnosis codes from the index hospitalization and 365 days prior were used to identify comorbidities including CKD, hypertension, and anemia (**Supplemental Table S2**) and the Deyo-modified Charlson Comorbidity Index (CCI) score (**Supplemental Table S3**). The ICD-10-CM diagnosis codes during index hospitalization were used to define dehydration with POA = Yes (E86.0), and sepsis, and acute respiratory failure (**Supplemental Table S2**).

### Outcomes

Primary outcomes were dialysis, in-hospital mortality, ICU admission, total LOS and costs, and ICU LOS and costs during index hospitalization, as previously described. Secondary outcomes, assessed during 30 days postdischarge (alive) from index hospitalization, were readmissions, readmission LOS and costs, outpatient visits and costs, in-hospital mortality, dialysis, and “new” dialysis, which excludes patients with dialysis during index hospitalization.

Admission to ICU was defined as the percentage of patients who had any ICU service charge during index hospitalization. Total LOS was hospital-reported LOS for the Index hospitalization. Total costs are the sum of all costs incurred during the index hospitalization. The ICU LOS was calculated as number of days with ICU room and board charges during the index hospitalization. ICU costs are calculated as the sum of all costs incurred on the days with ICU room and board charges. Cost data in the PHD are based on a micro-costing approach from a hospital perspective. The PHD uses a reconciliation process that allows for verification and validation of hospital reporting for the use of resources and cost. Data audits are performed, and if reported costs submitted do not match the hospital’s financial statement, Premier works with the hospital to correct the discrepancy.

### Statistical Analysis

Characteristics, clinical outcomes, LOS, and costs were compared using descriptive statistics, with *P* values from the χ^2^ test, *t* test, or Wilcoxon rank-sum test, as appropriate. Adjusted models included age, sex, race/ethnicity, CCI score, hospital characteristics (number of beds, teaching status, region, urban/rural), admission point of origin, admission type, medical vs surgical (categorized by Medicare Severity Diagnosis Related Group [MS-DRG]), primary payer, CKD, and ICU use, and, in a sensitivity analysis, sepsis. For clinical outcomes, logistic regression quantified unadjusted and multivariable-adjusted odds ratios (OR) and 95% CI.

Generalized linear model (GLM) regression with a log link and negative binomial distribution was used to quantify unadjusted and adjusted LOS ratios (eg, the ratio of LOS for patients with AKI vs no AKI) with 95% CIs. Unadjusted and adjusted mean cost ratios were quantified using a GLM with a log link and gamma distribution. For LOS and cost outcomes with many zeros (eg, ICU LOS), a 2-part (ie, “hurdle”) model was used.^38^ In a 2-part model, first, a logistic regression model estimated the probability of having any costs (eg, any ICU use).^38^ Second, for patients with any use, a GLM with log link quantified relative differences in LOS and costs (ie, LOS ratio [95% CI], using a negative binomial distribution], or cost ratio [95% CI; using a gamma distribution]).

Finally, the absolute difference in adjusted mean LOS and costs was calculated using the recycled prediction method. The recycled prediction method uses the fitted regression models to predict adjusted outcome values (eg, cost or LOS) for each participant, first, as if all patients had AKI and then as if all patients did not have AKI, with all other covariates at the levels for that participant. The mean difference between the 2 exposure groups (eg, AKI vs no AKI) is the difference in adjusted mean cost or LOS is the difference between the mean predicted LOS or costs when exposure is set to the first group minus mean predicted LOS or costs when exposure is set to the second group. To obtain 95% CIs of the mean difference, random bootstrap resampling with 1000 iterations was used to create a distribution where the 2.5th and 97.5th percentiles are the 95% lower and upper bound CIs, respectively.^39^

## RESULTS

### Study Cohort

**Figure 1.**
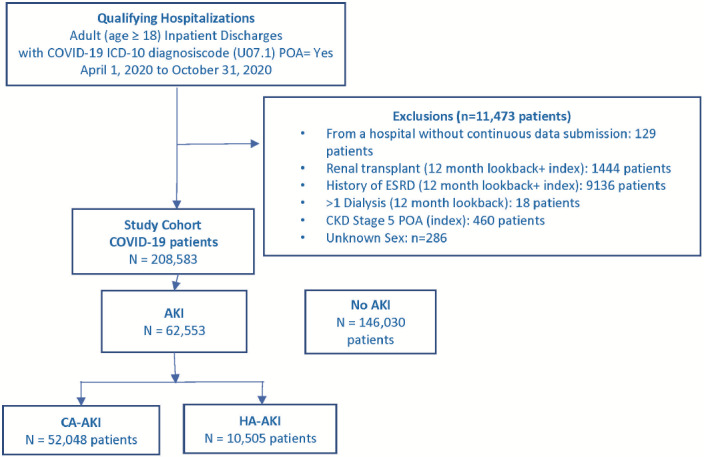
Patient Flow Diagram Abbreviations: AKI, acute kidney injury; CA-AKI, community-acquired AKI; CKD, chronic kidney disease; EGFR, estimated glomerular filtration rate; ESRD, end-stage renal disease; HA-AKI, hospital-acquired AKI; POA, present on admission.

During the study period, 220 056 adult patients with a COVID-19 ICD-10 diagnosis code were discharged from 829 US hospitals. Exclusion criteria removed 11 473 patients: 9136 for end-stage renal disease and 2337 for other exclusions, leaving 208 583 patients in the study cohort (**Figure 1**). Overall, the prevalence of AKI and CA-AKI were 30% and 25%, and the incidence of HA-AKI was 5%. Among the 49 990 (24%) of patients with ICU use, the corresponding AKI proportions were 52.1%, 37.3%, and 14.8%, and among the 158 593 (76%) patients without ICU use were 23.0%, 21.0%, and 2.0%, respectively. However, 58.4% (36 524/62 553) of AKI cases and 29.6% (3107/10 505) of HA-AKI cases occurred among non-ICU patients.

### Baseline Characteristics and Discharge Status

Patients with AKI, CA-AKI, and HA-AKI had higher mean age, were more likely to be men, Black, and in larger, urban, Northeast teaching hospitals; have emergency admissions, transfers from another facility (acute care or long-term), CKD, hypertension, diabetes, anemia, or higher mean CCI scores; and have dehydration POA, sepsis, or acute respiratory failure during hospitalization (**Table 1**, *P* < .005 for all vs no AKI). Patients with AKI, CA-AKI, and HA-AKI were also more likely to die or be discharged to hospice or a nursing or rehabilitation facility.

**Table 1.** Characteristics of Patients With COVID-19, Overall and by AKI Categories, Index Hospitalization (N = 208 583)

	**No AKI (n = 146 030)**	**AKI (n = 62 553)**	**CA-⁠AKI (n = 52 048)**	**HA-⁠AKI (n = 10 505)**
Age (y), mean ± SD	58.9 ± 18.6	69.3 ± 14.8	69.8 ± 14.9	67.1 ± 14.2
Age group (y), n (%)				
18-39	25 839 (17.7)	2425 (3.9)	2001 (3.8)	424 (4.0)
40-59	45 583 (31.2)	12 329 (19.7)	9888 (19.0)	2441 (23.2)
60-74	41 982 (28.7)	22 992 (36.8)	18 716 (36.0)	4276 (40.7)
≥75	626 (22.3)	807 (39.7)	443 (41.2)	3364 (32.0)
Gender, n (%)				
Male	69 252 (47.4)	37 252 (59.6)	30 863 (59.3)	6389 (60.8)
Race/ethnicity, n (%)				
Non-⁠Hispanic White	67 067 (45.9)	27 862 (44.5)	23 420 (45.0)	4442 (42.3)
Non-Hispanic Black	25 549 (17.5)	16 873 (27.0)	14 698 (28.2)	2175 (20.7)
Hispanic	34 084 (23.3)	9637 (15.4)	7366 (14.2)	2271 (21.6)
Other or unknown	19 330 (13.2)	8181 (13.1)	6564 (12.6)	1617 (15.4)
Primary payer, n (%)				
Medicare	60 690 (41.6)	40 599 (64.9)	34 491 (66.3)	6108 (58.1)
Medicaid	26 471 (18.1)	6951 (11.1)	5503 (10.6)	1448 (13.8)
Private insurance	42 837 (29.3)	11 031 (17.6)	8785 (16.9)	2246 (21.4)
Uninsured	6926 (4.7)	1462 (2.3)	1175 (2.3)	287 (2.7)
Other/unknown	9106 (6.2)	2510 (4.0)	2094 (4.0)	416 (4.0)
Hospital size, n (%)				
1-299	54 233 (37.1)	22 013 (35.2)	18 700 (35.9)	3313 (31.5)
300-499	43 288 (29.6)	19 137 (30.6)	16 028 (30.8)	3109 (29.6)
≥500	48 368 (33.1)	21 355 (34.1)	17 288 (33.2)	4067 (38.7)
Population served, n (%)				
Rural	17 479 (12.0)	6542 (10.5)	5597 (10.8)	945 (9.0)
Urban	128 551 (88.0)	56 011 (89.5)	46 451 (89.2)	9560 (91.0)
Teaching status, n (%)				
Non-teaching	77 636 (53.2)	30 297 (48.4)	25 416 (48.8)	4881 (46.5)
Teaching	68 394 (46.8)	32 256 (51.6)	26 632 (51.2)	5624 (53.5)
Region of hospital, n (%)				
Midwest	27 057 (18.5)	12 162 (19.4)	10 445 (20.1)	1717 (16.3)
Northeast	28 703 (19.7)	15 665 (25.0)	12 721 (24.4)	2944 (28.0)
South	70 875 (48.5)	28 130 (45.0)	23 663 (45.5)	4467 (42.5)
West	19 395 (13.3)	6596 (10.5)	5219 (10.0)	1377 (13.1)
Admission type, n (%)				
Elective	6933 (4.7)	1092 (1.7)	917 (1.8)	175 (1.7)
Emergency	118 975 (81.5)	54 207 (86.7)	45 214 (86.9)	8993 (85.6)
Urgent	18 747 (12.8)	6884 (11.0)	5614 (10.8)	1270 (12.1)
Trauma center	487 (0.3)	158 (0.3)	136 (0.3)	22 (0.2)
Unknown	888 (0.6)	212 (0.3)	167 (0.3)	45 (0.4)
Admission point of origin, n (%)				
Non-healthcare facility (eg, home)	119 463 (81.8)	48 729 (77.9)	40 395 (77.6)	8334 (79.3)
Clinic	7698 (5.3)	2907 (4.6)	2505 (4.8)	402 (3.8)
Transfer from acute care facility	13 346 (9.1)	6734 (10.8)	5424 (10.4)	1310 (12.5)
Transfer from long-term care facility	4389 (3.0)	3737 (6.0)	3347 (6.4)	390 (3.7)
Other/unknown	1134 (0.8)	446 (0.7)	377 (0.7)	69 (0.7)
MS-DRG categorization, n (%)				
Medical	138 390 (94.8)	58 530 (93.6)	49 586 (95.3)	8944 (85.1)
Surgical	7597 (5.2)	4013 (6.4)	2457 (4.7)	1556 (14.8)
History of comorbidities, n (%)				
Charlson Comorbidity Index	1.5 ± 1.9	3.0 ± 2.4	3.1 ± 2.4	2.7 ± 2.4
Charlson comorbidities				
Chronic kidney disease	12 413 (8.5)	26 334 (42.1)	23 249 (44.7)	3085 (29.4)
Hypertension	85 584 (58.6)	52 341 (83.7)	43 884 (84.3)	8457 (80.5)
Diabetes	50 941 (34.9)	33 320 (53.3)	27 710 (53.2)	5610 (53.4)
Anemia	26 886 (18.4)	23 166 (37.0)	18 688 (35.9)	4478 (42.6)
In-hospital complications				
Dehydration present on admission	11 822 (8.1)	14 982 (24.0)	13 929 (26.8)	1053 (10.0)
Sepsis	30 182 (20.7)	29 559 (47.3)	22 593 (43.4)	6966 (66.3)
Acute respiratory failure	73 521 (50.3)	36 895 (59.0)	30 664 (58.9)	6231 (59.3)
Discharge status				
Home	91 148 (62.4)	16 927 (27.1)	15 758 (30.3)	1169 (11.1)
Home health	16 398 (11.2)	6997 (11.2)	6120 (11.8)	877 (8.3)
Nursing or rehabilitation facility	19 421 (13.3)	13 222 (21.1)	11 189 (21.5)	2033 (19.4)
Transferred to an acute care hospital	696 (0.5)	262 (0.4)	229 (0.4)	33 (0.3)
Hospice	3141 (2.2)	3175 (5.1)	2801 (5.4)	374 (3.6)
Expired	8730 (6.0)	19 388 (31.0)	13 721 (26.4)	5667 (53.9)
Other/unknown	6496 (4.4)	2582 (4.1)	2230 (4.3)	352 (3.4)

For age and Charlson Comorbidity Index, results are reported as mean (SD) and compared using *t* test. Categorical variables are compared using χ^2^ test. *P* < .005 for all comparisons vs AKI. *P* < .005 for HA-AKI vs CA-AKI except diabetes (*P* = .76).

Abbreviations: AKI, acute kidney injury; CA-AKI, community-acquired AKI; HA-AKI, hospital-acquired AKI; MS-DRG, Medicare Severity Diagnosis Related Group.

In contrast, compared with CA-AKI, patients with HA-AKI had a slightly lower mean age (approximately 67 vs 70 years) and were less likely to be Black, have a history of CKD or hypertension, but were more likely to have a history of anemia, had a lower mean CCI, and had less dehydration POA, more sepsis, and slightly more acute respiratory failure (**Table 1**, *P* < .005 for all). Compared with CA-AKI, patients with HA-AKI were also slightly more likely to be in large, urban, teaching hospitals in the Northeast and more likely to die during index hospitalization.

### Clinical Outcomes During Index Hospitalization and 30-day Follow-up

During index hospitalization, the incidence of dialysis was 9.7% in AKI, 7.0% in CA-AKI, and 23.4% in HA-AKI patients (**Table 2**, *P* < .0001 for HA-AKI vs CA-AKI). Overall, 36.4% (76 019) of patients were admitted to the ICU, but patients with AKI, CA-AKI, and HA-AKI had much higher ICU use (35.8%-70.4% vs 16.4%) and in-hospital mortality (26.4%-53.9% vs 6.0%) than patients without AKI (**Table 2**, *P* < .0001 for all). During 30-day follow-up, patients with AKI, CA-AKI, and HA-AKI also had higher rates of readmission (8.7%-10.1% vs 6.8%), in-hospital mortality (1.1%-1.6% vs 0.7%), dialysis (0.6%-0.7% vs 0.1%), and “new” dialysis (0.3%-0.4% vs 0.1%), than patients without AKI (*P* < .005 for all), but less outpatient visits (*P* < .05) (**Table 2**). Furthermore, compared with CA-AKI, patients with HA-AKI had substantially higher ICU use (70.4% vs 35.8%) and in-hospital mortality (53.9% vs 26.4%) (⁠*P* < .0001 for both), but slightly lower readmissions (8.7% vs 10.1%, *P* = .003) and 30-day in-hospital mortality (1.1% vs 1.6%, *P* = .014) and similar 30-day outpatient visits and new dialysis (**Table 2**).

**Table 2.** Unadjusted Clinical Outcomes of Patients With COVID-19 by AKI Categories

	**No AKI (n=146 030)**	**AKI** **(n=62 553)**	**CA-AKI (n=52 048)**	**HA-AKI (n=10 505)**	***P* Values**			
					**AKI vs No AKI**	**HA-AKI** **vs No AKI**	**CA-AKI** **vs No AKI**	**HA-⁠AKI vs CA-AKI**
Outcomes during index hospitalization								
Dialysis, n (%)	84 (0.1)	6083 (9.7)	3630 (7.0)	2453 (23.4)	<.0001	<.0001	<.0001	<.0001
ICU admission, n (%)	23 961 (16.4)	26 029 (41.6)	18 631 (35.8)	7398 (70.4)	<.0001	<.0001	<.0001	<.0001
In-⁠hospital mortality, n (%)	8730 (6.0)	19 388 (31.0)	13 721 (26.4)	5667 (53.9)	<.0001	<.0001	<.0001	<.0001
Outcomes during 30 days postdischarge								
Patients surviving index hospitalization								
No. at risk	137 300	43 165	38 327	4838				
30-day readmission, n (%)	9280 (6.8)	4278 (9.9)	3856 (10.1)	422 (8.7)	<.0001	<.0001	<.0001	.0033
30-⁠day outpatient visits, n (%)	17 118 (12.5)	4930 (11.4)	4380 (11.4)	550 (11.4)	<.0001	0.0227	<.0001	.9022
30-day in-hospital mortality, n (%)	1023 (0.7)	668 (1.5)	613 (1.6)	55 (1.1)	<.0001	0.002	<.0001	.014
30-day dialysis, n (%)	119 (0.1)	252 (0.6)	216 (0.6)	36 (0.7)	<.0001	<.0001	<.0001	.1204
Patients surviving index hospitalization without dialysis								
No. at risk	137 131	41 387	37 171	4216				
30-day new dialysis, n (%)	118 (0.1)	148 (0.4)	136 (0.4)	12 (0.3)	<.0001	<.0001	<.0001	.4023

Continuous variables compared using Wilcoxon rank sum test. Categorical variables compared using χ^2^ test. “New” dialysis is dialysis during 30 days postdischarge among patients who survived index hospitalization with no dialysis.

Abbreviations: AKI, acute kidney injury; CA-AKI, community-acquired AKI; HA-AKI, hospital-acquired AKI; ICU, intensive care unit.

Odds of ICU use and in-hospital mortality during index hospitalization were higher for all AKI groups vs no AKI, but also for patients with HA-AKI vs those with CA-AKI, with adjusted ORs (aORs) of 4.1 for ICU use and 2.6 for in-hospital mortality (**Table 3**, *P* ≤ .001 for all). For outcomes during 30 days postdischarge, aORs for AKI, CA-AKI, and HA-AKI vs no AKI were 4.4 to 5.3 times higher for dialysis, and 2.4 to 2.8 times higher for new dialysis (**Table 3**, *P* ≤ .002 for all). In contrast, aORs for 30-day readmissions and in-hospital mortality were similar across AKI and no-AKI groups (**Table 3**).

**Table 3.** Multivariable-Adjusted Odds Ratios for Clinical Outcomes by AKI Categories Among COVID-19 Patients

	**AKI vs No AKI**		**CA-AKI vs No AKI**		**HA-AKI vs No AKI**		**HA-AKI vs CA-AKI**	
	**OR (95% CI)**	***P* Value**	**OR (95% CI)**	***P* Value**	**OR (95% CI)**	***P* Value**	**OR (95% CI)**	***P* Value**
Outcomes during index hospitalization								
ICU use (n = 49 990)								
Unadjusted	3.63 (3.55, 3.71)	<.0001	2.84 (2.78, 2.91)	<.0001	12.13 (11.61, 12.68)	<.0001	4.27 (4.08, 4.47)	<.0001
Adjusted^a^	3.70 (3.61, 3.79)	<.0001	2.87 (2.80, 2.95)	<.0001	11.81 (11.28, 12.37)	<.0001	4.11 (3.92, 4.31)	<.0001
In-hospital mortality (n = 28 118)								
Unadjusted	7.06 (6.87, 7.26)	<.0001	5.63 (5.47, 5.80)	<.0001	18.42 (17.63, 19.25)	<.0001	3.27 (3.13, 3.42)	<.0001
Adjusted^a^	3.52 (3.41, 3.63)	<.0001	2.88 (2.79, 2.98)	<.0001	7.61 (7.24, 8.01)	<.0001	2.64 (2.52, 2.78)	<.0001
Outcomes during 30 days postdischarge, among survivors of index hospitalization, n = 180 465								
30-day readmissions (n = 13 558)								
Unadjusted	1.52 (1.46, 1.58)	<.0001	1.54 (1.48, 1.61)	<.0001	1.32 (1.19, 1.46)	<.0001	0.85 (0.77, 0.95)	.0034
Adjusted^a^	1.03 (0.99, 1.07)	.1581	1.04 (1.00, 1.08)	.0825	0.96 (0.86, 1.06)	.4151	0.92 (0.83, 1.03)	.1349
30-day outpatient visits (n = 22 048)								
Unadjusted	0.91 (0.88, 0.94)	<.0001	0.91 (0.87, 0.94)	<.0001	0.90 (0.82, 0.99)	.0228	0.99 (0.90, 1.09)	.9022
Adjusted^a^	0.83 (0.79, 0.86)	<.0001	0.82 (0.79, 0.85)	<.0001	0.85 (0.77, 0.94)	.0008	1.04 (0.94, 1.14)	.4775
30-day in-hospital mortality (n = 1691)								
Unadjusted	2.09 (1.90, 2.31)	<.0001	2.17 (1.96, 2.39)	<.0001	1.53 (1.17, 2.01)	.0022	0.71 (0.54, 0.93)	.0145
Adjusted^a^	1.09 (0.98, 1.21)	.1149	1.10 (0.99, 1.23)	.0697	0.93 (0.70, 1.23)	.6001	0.84 (0.63, 1.11)	.2255
30-day dialysis (n = 371)								
Unadjusted	6.77 (5.44, 8.42)	<.0001	6.53 (5.22, 8.17)	<.0001	8.64 (5.95, 12.56)	<.0001	1.32 (0.93, 1.88)	.1215
Adjusted^a^	4.46 (3.50, 5.70)	<.0001	4.37 (3.41, 5.60)	<.0001	5.28 (3.53, 7.89)	<.0001	1.21 (0.84, 1.75)	.3134
30-day new dialysis^b^ (n = 266)								
Unadjusted	4.17 (3.27, 5.31)	<.0001	4.27 (3.33, 5.46)	<.0001	3.32 (1.83, 6.01)	<.0001	0.78 (0.43, 1.40)	.4035
Adjusted^a^	2.76 (2.11, 3.61)	<.0001	2.80 (2.13, 3.68)	<.0001	2.37 (1.28, 4.38)	.0060	0.85 (0.46, 1.54)	.5859

Abbreviations: AKI, acute kidney injury; CA-AKI, community-acquired AKI; HA-AKI, hospital-acquired AKI; ICU, intensive care unit; OR, odds ratio.

^a^Adjusted for age, sex, race/ethnicity, Charlson Comorbidity Index, hospital characteristics (number of beds, teaching status, region, urban/rural), admission point of origin, admission type, medical vs surgical (categorized by Medicare Severity Diagnosis Related Group), primary payer, chronic kidney disease, and ICU use.

^b^New dialysis is dialysis among patients without dialysis during index admission. Outcomes during 30-day follow-up are assessed among patients who survived index hospitalization.

### HRU During Index Hospitalization and 30-Day Follow-up

**Figure 2.**
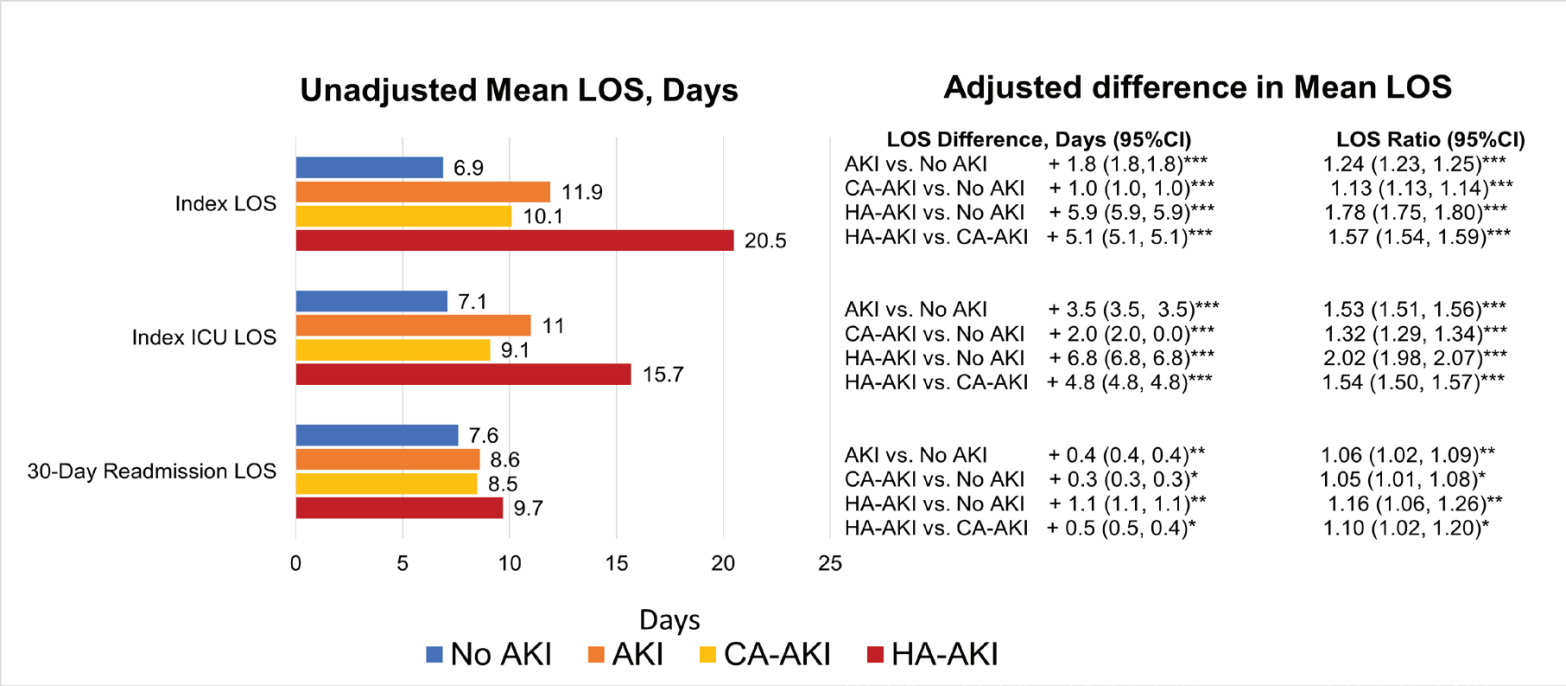
Mean LOS, Adjusted Mean Difference, and Adjusted LOS Ratio by AKI Categories Abbreviations: AKI, acute kidney injury; CA-AKI, community-acquired AKI; HA-AKI, hospital-acquired AKI; ICU, intensive care unit; LOS, length of stay. Adjusted models include these covariates: age, sex, race/ethnicity, Charlson Comorbidity Index, hospital characteristics (number of beds, teaching status, region, urban/rural), and admission point of origin, admission type, medical vs surgical (categorized by Medicare Severity Diagnosis Related Group codes), primary payer, ICU use, and chronic kidney disease. *P* < .0001 for all unadjusted comparisons vs no AKI; *P* < .005 for all unadjusted comparisons of HA-AKI vs CA-AKI. *P ≤ .02. **P < .005. ***P < .0001.

During index hospitalization, unadjusted and adjusted mean LOS was longer for all AKI groups vs no AKI, but also for HA-AKI vs CA-AKI (**Figure 2**, *P* < .001). Specifically, adjusted mean LOS was 1.8, 1.0, and 5.9 days longer, and on a ratio scale, 24% (LOS ratio, 1.24), 13%, and 78% longer for patients with AKI, CA-AKI, and HA-AKI vs no AKI, but also 5.1 days (57%) longer for HA-AKI vs CA-AKI (*P* < .0001 for all). Among patients with any ICU use, adjusted mean ICU LOS was 3.5 (53%),2.0 (32%), and 6.8 (202%) days longer in AKI, CA-AKI, and HA-AKI patients vs no AKI but also 4.8 days (54%) longer for HA-AKI vs CA-AKI (**Figure 2**, *P* < .0001). Finally, among readmitted patients, adjusted mean readmission LOS was longer (0.4, 0.3, and 1.1 days, respectively, or 5%-16%) for AKI, CA-AKI, and HA-AKI vs no AKI (**Figure 2**, *P* ≤ .02 for all) but also 0.5 days (10%) longer for HA-AKI vs CA-AKI (*P* = .02).

### Costs During Index Hospitalization and 30-Day Postdischarge Follow-up

**Figure 3.**
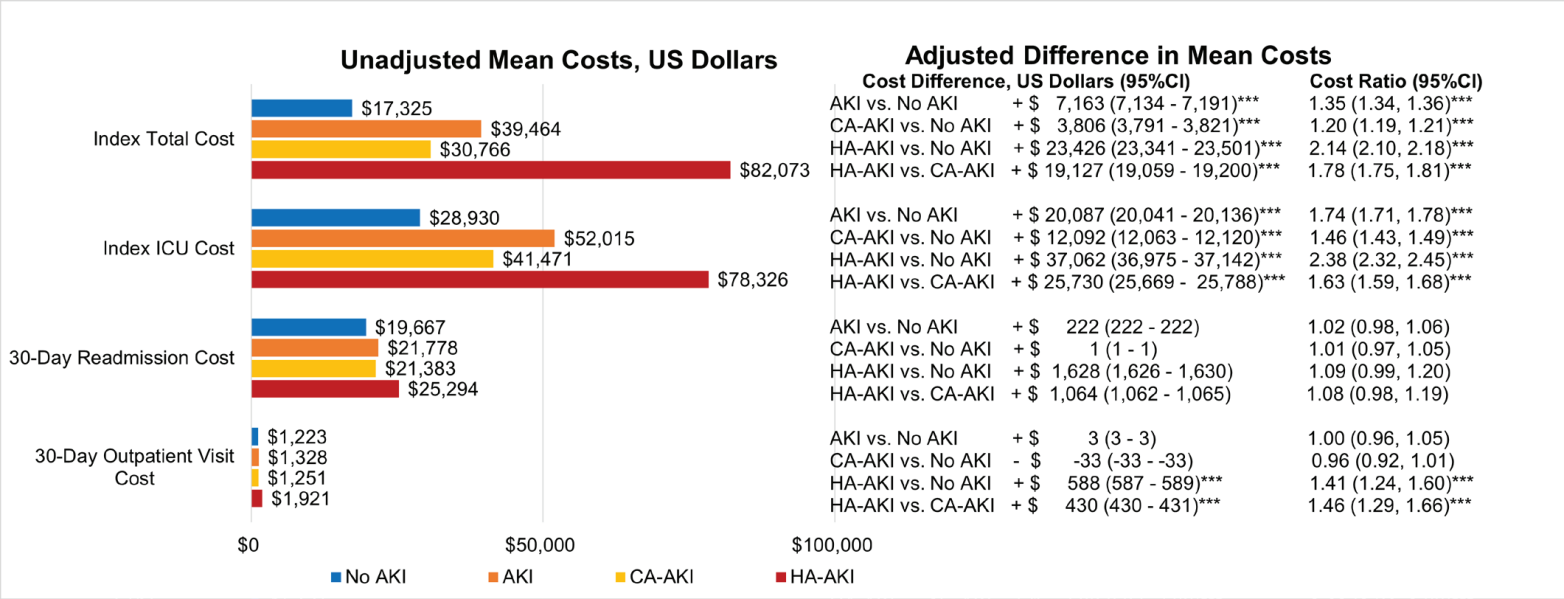
Mean Costs, Adjusted Mean Difference and Adjusted Cost Ratio by AKI Categories *P* < .0001 for all unadjusted comparisons vs no AKI; *P* < .001 for all unadjusted comparisons of HA-AKI vs CA-AKI. Adjusted models include these covariates: age, sex, race/ethnicity and Charlson Comorbidity Index, hospital characteristics (number of beds, teaching status, region, urban/rural), and admission point of origin, admission type, medical vs surgical (categorized by Medicare Severity Diagnosis Related Group codes), primary payer, ICU use, and chronic kidney disease. *P ≤ .02. **P < .005. ***P < .0001.

Unadjusted total and ICU costs during index hospitalization, and 30-day postdischarge readmission costs, were higher for all AKI groups than for patients without AKI, and all unadjusted costs were higher for HA-AKI vs CA-AKI (**Figure 3**, **Supplemental Table S4**, *P* < .001 for all). After adjusting for covariates, absolute and relative cost differences remained substantial. Specifically, adjusted mean total costs were $7163 (35%), $3806 (20%) and $23 426 (2.14 times) higher for AKI, CA-AKI, and HA-AKI vs no AKI, but also $19 127 (78%) higher for HA-AKI vs CA-AKI. Among ICU patients, adjusted mean ICU costs were $20 087 (74%), $12 092 (46%), and $37 062 (2.38 times) higher for AKI, CA-AKI, and HA-AKI vs no AKI, but also $25 730 (63%) higher for HA-AKI vs CA-AKI (*P* < .0001 for all). Among patients with 30-day outpatient visits, adjusted outpatient costs were significantly higher only for HA-AKI vs no AKI ($588, 41%) and HA-AKI vs CA-AKI ($430, 46%) (**Figure 3**, **Supplemental Table S4**, *P* < .001). In contrast, adjusted mean 30-day readmission costs among readmitted patients were similar for all AKI groups vs no AKI.

Finally, sensitivity analyses were conducted with models additionally adjusted for sepsis. All multivariable-adjusted results (aORs, LOS and cost ratios) were minimally affected with this additional adjustment (not shown).

## DISCUSSION

To our knowledge, this is the first study to report AKI-related costs among hospitalized COVID-19 patients. In our multicenter study of 208 583 US adults hospitalized with COVID-19 between April 1, 2020, and October 31, 2020, approximately 1 in 3 patients (30%) had AKI; 25% had CA-AKI (at admission), and 5% developed HA-AKI (during hospitalization). Adjusted for potential confounders, all AKI groups (AKI, CA-AKI, and HA-AKI) had higher odds of ICU use and in-hospital mortality, longer total and ICU LOS, and higher total and ICU costs during index hospitalization, and during 30-day postdischarge, had higher odds of dialysis and new dialysis, compared with patients with no AKI. Mean LOS was 13% and 78% longer and mean costs were 20% and 214% higher for CA-AKI and HA-AKI patients vs patients with no AKI. Notably, compared with CA-AKI, patients with HA-AKI had higher ICU use, in-hospital mortality, longer total and ICU LOS, and substantially higher costs during index hospitalization, but similar odds for outcomes during 30 days postdischarge. These results quantify the excess burden of AKI, especially HA-AKI, among COVID-19 patients, some of which might be reduced by early identification and appropriate management of COVID-19 patients at risk of AKI.

Our results for the prevalence of AKI (30% overall, 52.1% in ICU patients), odds of mortality (3.5 times higher for AKI vs no AKI), and mean LOS (11.9 days) are consistent with prior studies that extend past the first few months of the pandemic.^11,23^ Recent reviews and meta-analyses that include many studies of the early pandemic period reported the following ranges: 28% to 34%^11,14,15,40^ prevalence of overall AKI, 46% to 77% prevalence of AKI in ICU patients,^11,14,40^ and 2.55 to 23.09 for OR for mortality.^41^ Furthermore, in a study of AKI in COVID patients during all of 2020, HR (95% CI) for mortality was 3.8 (3.24-4.45), and mean LOS was 10.4 days.^42^ Among COVID patients admitted to UK hospitals in 2020, the adjusted OR (95% CI) for in-hospital mortality was 1.91 (1.82-2.01) for biochemically determined AKI (vs no AKI), 2.41 (2.20-2.64) for stage 2 AKI, and 3.50 (3.14-3.91) for stage 3 AKI.^43^

In our large multicenter study, HA-AKI and CA-AKI occurred in 5% and 25% of patients, and odds of mortality were 2.6 times higher, LOS was 1.6 times longer, and total costs were 1.8 times higher for HA-AKI vs CA-AKI. In 3 prior studies of COVID-19 patients, in-hospital mortality was 1.22 to 2.0 times higher for patients with HA-AKI vs CA-AKI.^20–22^ In 2 prior studies of COVID-19 patients, mortality was similar for patients with HA-AKI vs CA-AKI, but these studies were small (n = 448 UK patients^18^ and n = 1170 Mexican patients^19^) and during the early months of the pandemic when mortality rates were much higher. Length of stay was longer for HA-AKI vs CA-AKI in 2 studies^18,19^ and similar in 1 study.^21^ The results from our much larger study are consistent with studies among non-COVID patients, which found higher mortality,^16^ longer LOS, and higher costs^17^ for hospitalized patients with HA-AKI vs CA-AKI.

Study limitations include the use of hospital administrative data, which contains less clinical detail than electronic health records. The use of hospital-reported diagnosis procedure codes for diagnoses such as COVID-19 and AKI is well established,^34^ but ICD-10 codes do not identify AKI stage (ie, KDIGO stage 1, 2, 3). Using ICD codes to identify AKI has high specificity to identify AKI, especially higher-stage AKI, but lower sensitivity, especially for lower-stage AKI.^44^ Therefore, our study may have included a smaller proportion of stage 1 AKI, resulting in a more severe phenotype and lower prevalence of total AKI than if AKI were based on the changes in serum creatinine. However, this misclassification is likely to be small since the prevalence of AKI in our study is very similar to other recent studies that used KDIGO-defined serum creatinine changes to identify AKI.^14,43^ Another limitation is that events before (eg, history of dialysis) index hospitalization and 30-day postdischarge outcomes may be underestimated, since patient visits are linked only for visits to the same hospital system. However, we do have all data from the index hospitalizations, and relative differences (odds, LOS, and cost ratios) for 30-day outcomes should be minimally affected, since we expect underestimation to be nondifferential with respect to AKI type during index hospitalization. For example, the incidence of “new” dialysis was less than 1% of patients who survived index hospitalization without dialysis, but AKI, CA-AKI, and HA-AKI all had 2.4 to 2.8 times higher adjusted odds of new dialysis vs patients with no AKI. These results extend the literature showing longer-term adverse renal outcomes of COVID-19-associated AKI.^45,46^ Finally, a retrospective observational study design cannot prove causality, and results may be affected by unknown confounding. Our study does not have clinical severity scores such as SOFA or APACHE, or medication, but those may be more likely to be a confounder of mortality than cost. Regardless, this study adjusted for many important potential confounders, including age, CKD, and ICU use, and, given the magnitude of adjusted relative differences, it is unlikely that any unmeasured confounders would fully explain the associations between AKI and worse outcomes, particularly higher costs.

Our results have important implications for management of hospitalized COVID-19 patients. In hospitalized patients with and without COVID-19, studies demonstrate improved identification of patients at risk for AKI using risk scores,^47,48^ or other biomarkers,^49^ especially urinary biomarkers.^24–26,50,51^ Several studies, including randomized controlled trials, have shown that urinary biomarker–guided use of the KDIGO bundle resulted in lower incidence of stage 2/3 AKI^24–26^ and e-alert–guided use of the KDIGO bundle was associated with improved AKI recovery (reversal) within 7 days^27^ and with reduced LOS^28^ and costs.^30^ Still, adherence to best practices remains low in non-COVID patients with or at risk for moderate or severe AKI.^26,32,33^ Our results suggest that preventing AKI, especially HA-AKI, in COVID-19 patients may reduce adverse clinical outcomes, HRU, and costs, and therefore support implementation of recommended strategies for AKI prevention and management among hospitalized COVID-19 patients.

### Author Contributions

R.H.M., J.L.K., N.A.R., L.A.C., J.P.K., P.M., T.R., A.S., and J.T. participated in the design of the study, interpretation of data, and revision of the manuscript. L.C. created the analytic dataset with supervision and review by R.H.M. and N.A.R. R.H.M. performed the statistical analysis and wrote the first draft of the manuscript. R.H.M., J.L.K., N.A.R., L.A.C., J.P.K., P.M., T.R., A.S., and J.T. edited the manuscript and approved of the final version of the manuscript and agree to be accountable for all aspects of the work.

### Disclosures

R.H.M., N.A.R., and L.A.C. are full-time employees of Premier, Inc., which received payment from bioMerieux to conduct the study, and have no competing interests with respect to the study. R.H.M., N.A.R., and L.A.C. had access to the study data. J.T., P.M., J.P.K., A.S., and T.R. are full-time employees of bioMerieux, Inc., and P.M. and J.P.K. are inventors on patents assigned to bioMerieux and own shares in bioMerieux. J.L.K. reports consulting fees and research support from Astute-bioMerieux, Fresenius Medical, Mallinckrodt, Novartis, Guard Therapeutics, and the NIH and speakers bureau fees from NXStage Medical. The authors report no other conflicts of interest with this work.

## Supplementary Material

Online Supplementary Material
