# Supplementary material for: Outcomes, Healthcare Resource Utilization, and Costs of Overall, Community-Acquired, and Hospital-Acquired Acute Kidney Injury in COVID-19 Patients
Source: J Health Econ Outcomes Res. 2023 Feb 23;10(1):31–40. doi: 10.36469/001c.57651 (PMC9961448; doi:10.36469/001c.57651)
Supplement: Online Supplementary Material [file jheor_2023_10_1_57651_149975.pdf]

JHEOR

Journal of Health Economics and Outcomes Research

Online Supplementary Material

Outcomes, Healthcare Resource Utilization, and Costs of Overall, Community-Acquired, and Hospital-Acquired Acute Kidney Injury in COVID-19 Patients. *JHEOR*. **2023;10(1):31-40.** doi:10.36469/jheor.2023.57651

Supplemental Methods

Figure S1: Study Timeline

Table S1: ICD-10 Diagnosis and Procedure and CPT Codes for Conditions to Exclude, by Condition and Time Period

Table S2: ICD-10 Diagnosis Codes for Sepsis, Chronic Kidney Disease, Hypertension, and Anemia

Table S3: Charlson Comorbidity Index Diagnosis and Procedure Codes During Index Hospitalization and Prior 12 Months

Table S4: Unadjusted and Adjusted Absolute and Relative Cost Differences by AKI Categories Among Patients With COVID-19 During Index Hospitalization

This supplementary material has been provided by the authors to give readers additional information about their work.

This is an open-access article distributed under the terms of the Creative Commons Attribution 4.0 International License (CCBY-4.0). View this license's legal deed at http://creativecommons.org/licenses/by/4.0 and legal code at http://creativecommons.org/licenses/by/4.0/legalcode for more information.

SUPPLEMENTAL METHODS

Exposure: AKI, Community-acquired AKI (CA-AKI) and hospital-acquired AKI (HA-AKI)
Definition of present on admission (POA) indicator = Yes from ICD-10-CM Official Guidelines for Coding and Reporting FY 2022: "Present on admission is defined as present at the time the order for inpatient admission occurs—conditions that develop during an outpatient encounter, including emergency department, observation, or outpatient surgery, are considered as present on admission."

Source: Kidney Disease: Improving Global Outcomes (KDIGO) Acute Kidney Injury Work Group. KDIGO Clinical Practice Guideline for Acute Kidney Injury. *Kidney Int Suppl*. 2012;2(1):1-138.

Figure 1. Study Timeline

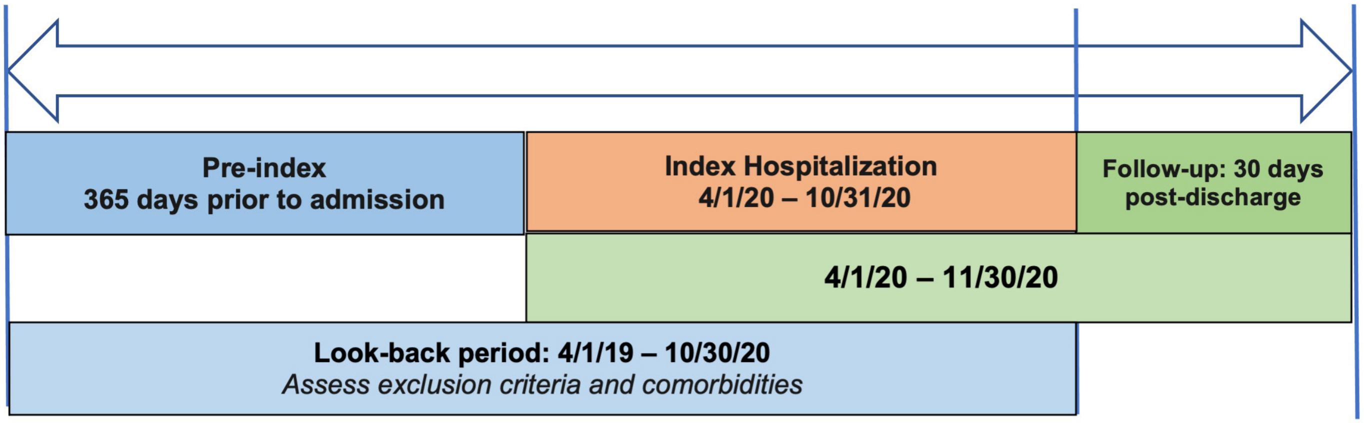

Table S1. ICD-10 Diagnosis and Procedure and CPT Codes for Conditions to Exclude, by Condition and Time Period

	ICD-10 Procedure Codes	CPT-4 Codes[a]
Extracorporeal membrane oxygenation (ECMO) procedure during index hospitalization		
ECMO	5A15223, 5A1522F, 5A1522G, 5A1522H	33946-33949, 33952, 33954, 33956, 33958, 33962, 33964, 33966, 33984, 33986-33989
End stage renal disease (ESRD) present on admission or during 12 mo prior to index hospitalization		
ESRD	N18.6	90957-90962, 90995, 90998
ESRD-related dialysis		90965, 90966, 90969, 90970, 90976-90985
Renal transplant during or 12 mo prior to index hospitalization		
Renal transplant	Z94.0	00868, 01990, 50300, 50320, 50323, 50325, 50327-50329, 50340, 50360, 50365, 50370, 50380
Complications of kidney transplant	T86.10, T86.11, T86.12, T86.13, T86.19	
CT scan of kidney transplant	BT2900Z, BT290ZZ, BT2910Z, BT291ZZ, BT29Y0Z, BT29YZZ, BT29ZZZ	
MRI of kidney transplant	BT39Y0Z, BT39YZZ, BT39ZZZ	
Ultrasonography of kidney transplant	BT49ZZ	
Transplantation, kidney, right; kidney, left	0TY0, 0TY1	
Dialysis-related diagnosis or procedures, ≥2 discharges during 12 mo prior to index hospitalization		
Care for renal dialysis	R88.0, Z49.01, Z49.02, Z49.31, Z49.32	90935, 90937, 90939-90945, 90947, 90988-90994, 90996, 90997, 90999
Patient noncompliance with renal dialysis	Z91.15	
Dependence on renal dialysis	Z99.2	
Complication following kidney dialysis	T81.502A, T81.502D, T81.502S, T81.512A, T81.512D, T81.512S,<br>T81.522A, T81.522D, T81.522S,<br>T81.532A, T81.532D, T81.532S,<br>T81.592A, T81.592D, T81.592S	
Mechanical complication of vascular dialysis catheter	T82.41XA, T82.41XD, T82.41XS<br>T82.42XA, T82.42XD, T82.42XS<br>T82.43XA, T82.43XD, T82.43XS<br>T82.49XA, T82.49XD, T82.49XS	
Mechanical complication of intraperitoneal dialysis catheter	T85.611A, T85.611D, T85.611S,<br>T85.621A, T85.621D, T85.621S<br>T85.631A, T85.631D, T85.631S<br>T85.691A, T85.691D, T85.691S	
Infection/inflammatory reaction due to peritoneal dialysis catheter	T85.71XA, T85.71XD, T85.71XS	
Failure of sterile procedures during kidney dialysis or other perfusion	Y62.2	
Kidney dialysis as cause of abnormal reaction of patient or later complication	Y84.1	
Cloudy dialysis effluent	R88.0	
Radiography of dialysis shunt/fistula<br>Fluoroscopy of dialysis shunt/fistula	B50W0ZZ, B50W1ZZ, B50WYZZ<br>B51W0ZA, B51W0ZZ, B51W1ZA, B51W1ZZ,<br>B51WYZA, B51WYZZ, B51WZZA, B51WZZZ	
Performance of urinary filtration<br>Irrigation of peritoneal cavity using dialysate	5A1D70Z, 5A1D80Z, 5A1D90Z,<br>3E1M39Z	

Table S2. ICD-10 Diagnosis Codes for Sepsis, Chronic Kidney Disease, Hypertension, and Anemia

Description	ICD-10 Codes
Sepsis during index hospitalization	
Salmonella sepsis	A02.1
Septicemic plague	A20.7
Anthrax sepsis	A22.7
Melliodosis sepsis	A24.1
Erysipelothrix sepsis	A26.7
Sepsis due to *Listeria monocytogenes*	A32.7
Meningococcemia, unspecified (includes sepsis)	A39.4
Sepsis due to streptococcus	A40.x
Other sepsis	A41.x
Actinomycotic sepsis	A42.7
Gonococcal sepsis	A54.86
Herpesviral sepsis or disseminated herpesviral disease	B00.7
Candidal sepsis	B37.7
Septic arterial embolism	I76
Sepsis following incomplete spontaneous abortion	O03.37
Sepsis following complete spontaneous abortion	O03.87
Sepsis following pregnancy termination	O04.87
Sepsis following failed pregnancy termination	O07.37
Sepsis following ectopic and molar pregnancy	O08.82
Sepsis during labor	O75.3
Puerperal sepsis	O85
Sepsis following obstetrical procedure	O86.04
Severe sepsis	R65.20
Septic shock	R65.21
Bloodstream infection due to central venous catheter (includes sepsis)	T80.211x
Sepsis following a procedure	T81.44x
Sepsis following immunization	T88.0
Chronic kidney disease during index hospitalization or prior 12 mo	D63.1, I12.x, I13.x, N18.x
Hypertension during index hospitalization or prior 12 mo	I10, I11.x, I12.x, I13.x, I15.x, I16.x
Anemia during index hospitalization or prior 12 mo	
Iron deficiency anemias	D50.x
Other deficiency anemias	D51.x, D52.x, D53.x
Hereditary hemolytic anemias	D55.x, D56.x, D57.x, D58.x
Acquired hemolytic anemias	D59.x
Aplastic anemia and other bone marrow failure syndromes	D60.x, D61.x, D62.x, D63.x, D64.x
Other and unspecified anemias	Codes are included above
Acute respiratory failure during index hospitalization	
Acute respiratory failure	J96.0, J96.00, J96.01, J96.02
Acute and chronic respiratory failure	J96.2, J96.20, J96.21, J96.22
Respiratory failure, unspecified	J96.9, J96.90, J96.91, J96.92

Table S3. Charlson Comorbidity Index Diagnosis and Procedure Codes During Index Hospitalization and Prior 12 Months

**Comorbidity**	**ICD-10 Codes**
Myocardial infarction	I21.09, I21.19, I21.11, I21.29, I21.3, I21.41, I21.42, I25.2
Congestive heart failure	I50.9, I50.1, I50.20, I50.21, I50.22, I50.23, I50.30, I50.31, I50.32, I50.33, I50.40, I50.41, I50.42, I50.43, I50.9
Peripheral vascular disease	I73.9, I71.00, I71.01, I71.02, I71.03, I71.1, I71.2, I71.3, I71.4, I71.5, I71.6, I71.8, I71.9, I96, Z95.828, 04RK07Z, 04RK0JZ, 04RK0KZ, 04RK47Z, 04RK4JZ, 04RK4KZ, 04RL07Z, 04RL0JZ, 04RL0KZ, 04RL47Z, 04RL4JZ, 04RL4KZ, 04RM07Z, 04RM0JZ, 04RM0KZ, 04RM47Z, 04RM4JZ, 04RM4KZ, 04RN07Z, 04RN0JZ, 04RN0KZ, 04RN47Z, 04RN4JZ, 04RN4KZ, 04RP07Z, 04RP0JZ, 04RP0KZ, 04RP47Z, 04RP4JZ, 04RP4KZ, 04RQ07Z, 04RQ0JZ, 04RQ0KZ, 04RQ47Z, 04RQ4JZ, 04RQ4KZ, 04RR07Z, 04RR0JZ, 04RR0KZ, 04RR47Z, 04RR4JZ, 04RR4KZ, 04RS07Z, 04RS0JZ, 04RS0KZ, 04RS47Z, 04RS4JZ, 04RS4KZ, 04RT07Z, 04RT0JZ, 04RT0KZ, 04RT47Z, 04RT4JZ, 04RT4KZ, 04RU07Z, 04RU0JZ, 04RU0KZ, 04RU47Z, 04RU4JZ, 04RU4KZ, 04RV07Z, 04RV0JZ, 04RV0KZ, 04RV47Z, 04RV4JZ, 04RV4KZ, 04RW07Z, 04RW0JZ, 04RW0KZ, 04RW47Z, 04RW4JZ, 04RW4KZ, 04RY07Z, 04RY0JZ, 04RY0KZ, 04RY47Z, 04RY4JZ, 04RY4KZ
Cerebrovascular disease	I60.x, I61.x, I62.x, I63.x, I65.x, I66.x, I67.x, I68.x, I69.x, G45.x
Dementia	F03.90, F01.50, F01.51, F03.91, F02.80, F02.81
Chronic pulmonary disease	J40, J41.0, J41.1, J44.9, J44.0, J41.8, J42, J43.9, J45.20, J45.22, J45.21, J44.1, J45.990, J45.991, J45.909, J45.998, J45.902, J45.901, J47.9, J47.1, J67.0, J67.1, J67.2, J67.3, J67.4, J67.5, J67.6, J67.7, J67.8, J67.9, J60, J61, J62.8, J63.0, J63.1, J63.2, J63.3, J63.4, J63.5, J63.6, J66.0, J66.1, J66.2, J66.8, J64, J68.4
Rheumatic disease	M32.10, M34.0, M34.1, M34.9, M33.20, M06.9, M05.00, M05.30, M05.60, M06.1, M05.10, M35.3
Peptic ulcer disease	K25.0, K25.1, K25.2, K25.3, K25.4, K25.5, K25.6, K25.7, K25.9, K26.0, K26.1, K26.2, K26.3, K26.4, K26.5, K26.6, K26.7, K26.9, K27.0, K27.1, K27.2, K27.3, K27.4, K27.5, K27.6, K27.7, K27.9, K28.0, K28.1, K28.2, K28.3, K28.4, K28.5, K28.6, K28.7, K28.9
Mild liver disease	K70.30, K73.9, K73.0, K75.4, K73.2, K73.8, K74.0, K74.60, K74.69, K74.3, K74.4, K74.5
Diabetes without chronic complication	E11.9, E10.9, E13.9, E11.65, E10.65, E13.65, E10.1x, E11.1x, E13.1x, E11.0x, E13.0x, E11.64x, E10.64x, E13.64x
Diabetes with chronic complication	E10.2x, E10.3x, E10.4x, E10.5x, E10.61x, E10.62x, E10.63x, E10.69x, E10.8, E11.2x, E11.3x, E11.4x, E11.5x, E11.61x, E11.62x, E11.63x, E11.69, E11.8, E13.2x, E13.3x, E13.4x, E13.5x, E13.61x, E13.62x, E13.63x, E13.69, E13.8
Hemiplegia or paraplegia	G82.20, G81.00, G81.01, G81.02, G81.03, G81.04, G81.10, G81.11, G81.12, G81.13, G81.14, G81.90, G81.91, G81.92, G81.93, G81.94
Moderate or severe renal disease	N03.2, N03.3, N03.5, N03.8, N08, N03.9, N05.9, N05.2, N05.5, N17.1, N17.2, N18.1, N18.2, N18.3, N18.4, N18.5, N18.6, N18.9, N19, N25.0, N25.1, N25.81, N25.89, N25.9
Any malignancy, including lymphoma and leukemia, except malignant neoplasm of skin	C00.x, C01, C02.x, C03.x, C04.x, C05.x, C06.x, C07, C08.x, C09.x, C10.x, C11.x, C12, C13.x, C14.x, C15.x, C16.x, C17.x, C18.x, C19, C20, C21.x, C22.x, C23, C24.x, C25.x, C26.x, C30.x, C31.x, C32.x, C33, C34.x, C37, C38.x, C39.x, C40.x, C41.x, C45.x, C46.x, C47.x, C48.x, C49.x, C50.x, C51.x, C52, C53.x, C54.x, C55, C56.x, C57.x, C58, C60.x, C61, C62.x, C63.x, C64.x, C65.x, C66.x, C67.x, C68.x, C69.x, C70.x, C71.x, C72.x, C73, C74.x, C75.x, C7A.x, C7B.x, C76.x, C77.x, C78.x, C79.x, C80.x, C81.x, C82.x, C83.x, C84.x, C85.x, C86.x, C88.x, C90.x, C91.x, C92.x, C93.x, C94.x, C95.x, C96.x, D00.x, D01.x, D02.x, D03.x, D05.x, D06.x, D07.x, D09.x
Moderate or severe liver disease	I85.01, I85.00, I85.11, I85.10, K72.90, K72.91, K76.6, K76.7, K72.10, K72.90
Metastatic solid tumor	C77.0, C77.1, C77.2, C77.3, C77.4, C77.5, C77.8, C77.9, C78.00, C78.1, C78.2, C78.39, C78.4, C78.5, C78.6, C78.7, C78.89, C79.00, C79.11, C79.19, C79.2, C79.31, C79.32, C79.49, C79.51, C79.52, C79.60, C79.70, C79.81, C79.82, C79.89, C80.0, C80.1
AIDS/HIV	B20

Table S4. Unadjusted and Adjusted Absolute and Relative Cost Differences by AKI Categories Among Patients With COVID-19 During Index Hospitalization

Cost Difference	AKI vs No AKI			CA-AKI vs No AKI			HA-AKI vs No AKI			HA-AKI vs CA-AKI		
	Absolute Difference	Relative Difference		Absolute Difference	Relative Difference		Absolute Difference	Relative Difference		Absolute Difference	Relative Difference	
		Ratio (95% CI)	*P* Value		Ratio (95% CI)	*P* Value		Ratio (95% CI)	*P* Value		Ratio (95% CI)	*P* Value
Index hospitalization cost (n = 208 583)												
Unadjusted	$22 139	2.28 (2.26, 2.30)	<.0001	$13 441	1.78 (1.76, 1.79)	<.0001	$64 748	4.74 (4.64, 4.83)	<.0001	$51 307	2.67 (2.61, 2.72)	<.0001
Adjusted	$7163	1.35 (1.34, 1.36)	<.0001	$3806	1.20 (1.19, 1.21)	<.0001	$23 426	2.14 (2.10, 2.18)	<.0001	$9127	1.78 (1.75, 1.81)	<.0001
Index hospitalization ICU cost (n = 49 990)												
Unadjusted	$23 085	1.80 (1.76, 1.83)	<.0001	$12 541	1.43 (1.40, 1.46)	<.0001	$49 396	2.71 (2.63, 2.79)	<.0001	$36 855	1.89 (1.83, 1.95)	<.0001
Adjusted	$20 087	1.74 (1.71, 1.78)	<.0001	$12 092	1.46 (1.43, 1.49)	<.0001	$37 062	2.38 (2.32, 2.45)	<.0001	$25 730	1.63 (1.59, 1.68)	<.0001
Cost differences during 30-day follow-up, among survivors of index hospitalization (n = 180,465)												
30-day readmission cost (n = 13 558)												
Unadjusted	$111	1.11 (1.07, 1.15)	<.0001	$1716	1.09 (1.05, 1.13)	<.0001	$5627	1.29 (1.17, 1.42)	<.0001	$3911	1.18 (1.07, 1.31)	.0008
Adjusted	$222	1.02 (0.98, 1.06)	.3696	$1	1.01 (0.97, 1.05)	.6445	$1628	1.09 (0.99, 1.20)	.0714	$1064	1.08 (0.98, 1.19)	.1132
30-day outpatient cost (n = 22 048)												
Unadjusted	$105	1.09 (1.04, 1.14)	.0004	$28	1.02 (0.98, 1.07)	.3555	$698	1.57 (1.39, 1.77)	<.0001	$670	1.54 (1.35, 1.74)	<.0001
Adjusted	$3	1.00 (0.96, 1.05)	.8761	$(33)	0.96 (0.92, 1.01)	.1369	$588	1.41 (1.24, 1.60)	<.0001	$430	1.46 (1.29, 1.66)	<.0001

Abbreviations: AKI, acute kidney injury; CA-AKI, community-acquired AKI; HA-AKI, hospital-acquired AKI.
